# Left ventricular diastolic function assessed by speckle tracking echocardiography in patients with left ventricular aneurysm

**DOI:** 10.1007/s10554-024-03201-z

**Published:** 2024-07-25

**Authors:** Olena Nemchyna, Natalia Solowjowa, Yuriy Hrytsyna, Michael Dandel, Nicolas Merke, Jan Knierim, Felix Schoenrath, Isabell Anna Just, Felix Hennig, Felix Hohendanner, Volkmar Falk, Christoph Knosalla

**Affiliations:** 1 Department of Cardiothoracic and Vascular Surgery, Deustches Herzzentrum der Charité, Augustenburger Platz 1, 13353 Berlin, Germany; 2 Charité – Universitätsmedizin Berlin, Corporate Member of Freie Universität Berlin, Humboldt-Universität zu Berlin, and Berlin Institute of Health, Charitéplatz 1, 10117 Berlin, Germany; 3 DZHK (German Centre for Cardiovascular Research), Partner Site Berlin, Hessische Straße 3-4, 10115 Berlin, Germany; 4 Department of Health Science and Technology, ETH Zurich, Translational Cardiovascular Technology, LFW C 13.2, Universitätstrasse 2, 8092 Zurich, Switzerland; 5 Cardio Centrum Berlin, Unter den Linden 21, 10117 Berlin, Germany; 6 Sana Paulinenkrankenhaus, Dickensweg 25-39, 14055 Berlin, Germany; 7 Department of Cardiology, Angiology and Intensive Care MedicineDeutsches Herzzentrum der Charité, Augstenburger Platz 1, 13353 Berlin, Germany

**Keywords:** Surgical ventricular repair, Surgical ventricular restoration, Left ventricular aneurysm, Speckle-tracking echocardiography, Strain rate, Outcome study

## Abstract

**Abstract:**

Speckle-tracking echocardiography (STE) parameters are an integral part of the assessment of left ventricular (LV) function. We aimed to evaluate established and novel STE parameters of LV diastolic function and their prognostic role in patients with LV anteroapical aneurysm undergoing surgical ventricular repair (SVR). We retrospectively examined the data of 137 patients with anteroapical LV aneurysm who underwent SVR. In 27 patients, the correlation of STE parameters with invasive hemodynamic parameters was evaluated. Preoperative echocardiographic parameters were assessed for their association with outcome, defined as all-cause mortality, LV assist device implantation, or heart transplantation. The late diastolic strain rate (GLSRa) showed a stronger correlation with mean pulmonary artery pressure (*r* =  − 0.75, *p* < 0.001) than all other parameters. GLSRa was also significantly correlated with mean pulmonary capillary wedge pressure and LV end-diastolic pressure. In the multivariate model, GLSRa and the ratio of early diastolic filling velocity to GLSRa demonstrated incremental prognostic value in addition to clinical and echocardiographic parameters. Patients with GLSRa < 0.59 s^−1^ had significantly shorter event-free survival than those with GLSRa > 0.59 s^−1^ (6.7 vs. 10.9 years, *p* < 0.001). Peak reservoir left atrial strain showed a weaker association with hemodynamic parameters and outcome compared to GLSRa. In patients with LV aneurysm, late diastolic strain rate and left atrial strain can be used for the assessment of LV diastolic function and have a predictive value for the outcome after surgical ventricular restoration.

**Graphical abstract:**

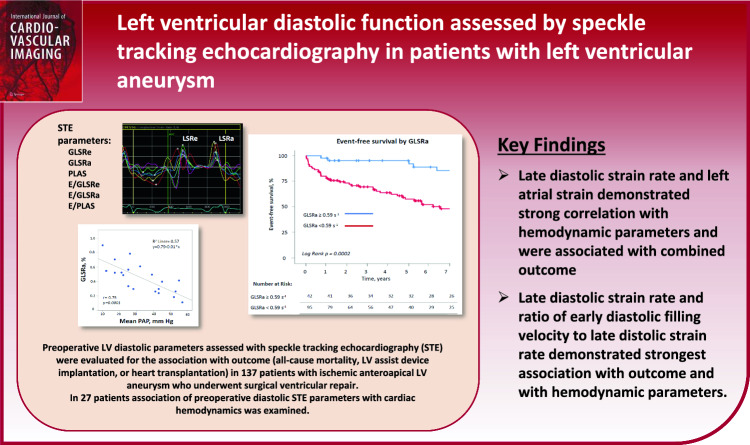

**Supplementary Information:**

The online version contains supplementary material available at 10.1007/s10554-024-03201-z.

## Introduction

Assessment of left ventricular (LV) diastolic function by echocardiography is of paramount importance for the evaluation of cardiac function and patient prognosis [1]. Increases in LV filling pressure and LV myocardial stiffness can be estimated indirectly by a number of echocardiographic parameters, including those obtained using speckle-tracking echocardiography (STE) [1, 2]. In previous studies, peak reservoir left atrial strain (PLAS), LV longitudinal diastolic strain rate measured at isovolumic relaxation time (SR_IVRT_) and during early diastole of the left ventricle demonstrated an association with increased LV filling pressure [3–5] and demonstrated prognostic value in patients with heart failure (HF) [6]. Although many echocardiographic parameters of LV diastolic function have been validated against invasive assessment of LV filling pressure [7, 8], there is still a lack of data in terms of their diagnostic and prognostic significance for various groups of patients.

Patients with LV aneurysms represent a specific population with advanced regional LV remodeling. LV remodeling is the enlargement of the left ventricle, a change in its geometry and an increase in wall stress, which progressively impairs LV systolic and diastolic function and clinically results in the progression of heart failure [9]. Surgical ventricular restoration (SVR) aims to reduce LV volume and prevent the progression of remodeling by removing extensive scar tissue, restoring LV geometry and further reducing wall stress [10]. Several factors were identified as predictors for worse outcome after SVR, including severe dilatation of the left ventricle, severe impairment of ejection fraction (EF), severe functional mitral regurgitation and advanced diastolic dysfunction [11–15]. Currently, there are no clear recommendations to guide the decision-making process for SVR in patients with an LV aneurysm. Therefore, additional criteria are needed to improve procedural planning, patient selection and outcome prediction after SVR.

The aim of this study was to evaluate various echocardiographic parameters of LV diastolic function, including those obtained with STE, in relation to cardiac hemodynamics in patients with LV anteroapical aneurysm and their association with outcomes after surgical ventricular restoration.

## Methods

### Study population and outcome

**Fig. 1:**
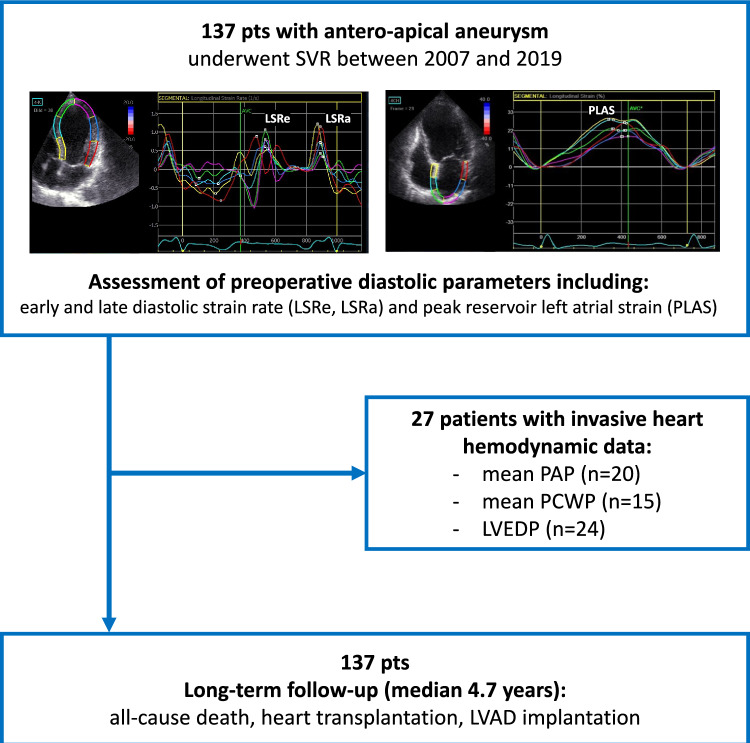
Study flow chart

We retrospectively evaluated data from 137 consecutive patients with postischemic anteroapical LV aneurysms who underwent SVR at our center between November 2007 and January 2019 and for whom comprehensive preoperative transthoracic echocardiography suitable for STE analysis and for conventional assessment of LV diastolic function by transmitral flow was available (Fig. 1). We did not include patients with overall poor image quality, with inadequate speckle tracking in ≥ 2 adjacent segments within an apical view, or with < 3 apical views per study. Patients were followed up for the combined outcome: all-cause death, LV assist device (LVAD) implantation, or heart transplantation. Mortality data were obtained from the National Death Index; data on heart transplantation and LVAD implantation were derived from medical records and telephone contacts. The study was performed according to the principles of the Declaration of Helsinki and approved by the local ethics committee (EA2/177/20).

### Surgical technique

All SVR procedures were performed using a median sternotomy approach using cardiopulmonary bypass. Concomitant coronary artery bypass surgery was performed in 72.3% of patients, and mitral valve repair/replacement was performed in 23.4%. A modified Dor procedure without a patch with one or more Fontan sutures along the aneurysm perimeter [16, 17] was performed in most cases. Fifteen patients (10.9%) were operated on using an endoventricular circular patch plasty [10], and thrombectomy was performed in 31 patients (22.6%) (Table 1).

**Table 1:** Patient baseline characteristics and univariate analysis for prediction of combined outcome

Parameter	All patients			
	*N* = 137	HR adjusted for age and sex	95% CI	*p*-value
Age, years (sex adjusted HR for 10 years)	60.8 ± 11	1.3	1.01–1.7	0.042
Women/Men (age adjusted HR for male sex)	34/103 (24.8/75.2)	0.86	0.49–1.5	0.61
BMI, kg/m^2^	27 [24; 31]	1.01	0.96–1.1	0.62
Diabetes mellitus	36 (26.3)	1.58	0.9–2.8	0.11
Arterial hypertension	98 (71.5)	1.28	0.69–2.4	0.43
Paroxysmal atrial fibrillation	13 (9.5)	2.9	1.4–6.3	0.006
Chronic kidney disease, stage ≥ 3	25 (18.2)	1.9	1.06–3.5	0.032
Plasma creatinine, mg/dL (HR for 0.1 mg/dL)	1.02 [0.87; 1.27]	1.06	1.02–1.1	0.009
NYHA functional class (HR for NYHA III/IV)				
I	5 (3.6)	1.32	0.4–4.3	0.65
II	10 (7.3)			
III	110 (80.3)			
IV	12 (8.8)			
Time since MI, years	2.8 [0.3; 12.6]	1.05	1.01–1.1	0.007
Previous heart surgery	8 (5.8)	2.6	1.0–6.8	0.05
*Intraoperative variables*				
Endoventricular patch	15 (10.9)	0.58	0.23–1.5	0.25
Thrombectomy	31 (22.6)	1.13	0.56–2.3	0.74
CABG	99 (72.3)	0.91	0.5–1.7	0.75
MV repair/replacement	32 (23.4)	1.6	0.9–2.7	0.13
AV replacement	7 (5.1)	1.5	0.6–3.8	0.39
Cross-clamp time, min	73 [51; 100]	1.004	1–1.05	0.3
Perfusion time, min	122 [93; 160]	1.003	1.0–1.01	0.19
*Echocardiography*				
LV shape type:				
1 (aneurysmal)	42 (30.7)	–	–	–
2 (intermediate)	79 (57.7)	1.9	0.97–3.6	0.064
3 (globally akinetic)	16 (11.7)	5.1	2.2–11.5	0.0001
LV EDDI, cm/m^2^	3.0 [2.7; 3.4]	1.8	1.1–3.1	0.024
LV ESDI, cm/m^2^	2.4 [2.0; 2.7]	2.3	1.4–3.7	0.001
LV EDVI, mL/m^2^ (HR for10 mL/m^2^)	104 [84; 134]	1.1	1.02–1.2	0.011
LV ESVI, mL/m^2^ (HR for10 mL/m^2^)	69[53; 96]	1.12	1.03–1.2	0.007
SI (HR for 0.1)	0.59 ± 0.1	1.63	1.2–2.3	0.005
LV EF, %	34 ± 9	0.96	0.93–0.99	0.004
LV FS, %	23 [15; 29]	0.95	0.92–0.98	0.001
LV SVI_LVOT_, mL/m^2^	27 [22; 33]	1.0	0.96–1.04	0.81
LV mass index, g/m^2^ (HR for 20 g/m^2^)	139 [115; 163]	1.19	1.04–1.35	0.01
LAVI, mL/m^2^(HR for10 mL/m^2^)	41 [33; 53]	1.2	1.02–1.4	0.03
TAPSE, mm	18 ± 4	0.96	0.9–1.03	0.23
E, m/s	0.68 [0.54; 0.83]	3.3	1.3–8.7	0.014
A, m/s	0.63 [0.41; 0.83]	0.41	0.14–1.2	0.09
E/A	1.07 [0.67; 1.95]	1.26	1.01–1.6	0.039
DTE, ms	173 [138; 228]	1.0	1.0–1.001	0.15
Diastolic dysfunction:				
Grade 1	60 (43.8)	–	–	–
Grade 2	31 (22.6)	1.57	0.78–3.2	0.21
Grade 3	46 (33.6)	2.26	1.22–4.2	0.009
MR 2 +	35 (25.5)	1.95	1.05–3.6	0.036
*STE*				
GLS, %	− 5.5 ± 3	1.11	1.02–1.2	0.03
GLSRs, s^−1^	− 0.53 [− 0.64;− 0.45]	4.1	0.7–24	0.12
GLSRe, s^−1^	0.6 [0.43; 0.77]	0.3	0.1–1.0	0.053
GLSRa, s^−1^	0.49 [0.34; 0.62]	0.06	0.01–0.24	< 0.0001
E/GLSRe, m	1.12 [0.84; 1.5]	1.06	1.01–1.1	0.02
E/GLSRa, m	1.38 [0.88; 2.23]	1.38	1.2–1.6	< 0.0001
BLS, %	− 9.2 ± 3.3	1.14	1.05–1.2	0.002
BLSRs, s^−1^	− 0.71 [− 0.8; − 0.58]	5.7	0.9–31	0.07
BLSRe, s^−1^	0.79 [0.62; 1.01]	0.63	0.02–1.7	0.37
BLSRa, s^−1^	0.64 [0.43; 0.87]	0.13	0.05–0.37	0.0001
E/BLSRe, m	0.85 [0.67; 1.14]	1.5	1.2–1.9	0.001
E/BLSRa, m	0.99 [0.65; 1.74]	1.3	1.2–1.5	< 0.0001
PLAS, %	17.9 [12; 24]	0.96	0.92–0.99	0.03
E/PLAS, cm/s/%	3.6 [2.4; 5.8]	1.08	1.04–1.12	0.0001

Data are presented as the mean ± SD, median [1. and 3. quartiles], or *n* (%)

*A* late diastolic filling velocity, *AV* aortic valve, *BMI* body mass index, *BLS* basal longitudinal strain, *BLSRa* basal late diastolic longitudinal strain rate, *BLSRe* basal early diastolic longitudinal strain rate, *BLSRs* basal systolic longitudinal strain rate, *CABG* coronary artery bypass graft, *CI* confidence interval, *DTE* early mitral flow deceleration time, *GLS* global longitudinal strain, *GLSRa* global late diastolic longitudinal strain rate, *GLSRe* global early diastolic longitudinal strain rate, *GLSRs* global systolic longitudinal strain rate, *E* early diastolic filling velocity, *EDDI* end-diastolic diameter index, *EDVI* end-diastolic volume index, *EF* ejection fraction, *ESDI* end-systolic diameter index, *ESVI* end-systolic volume index, *FS* fractional shortening, *HR* hazard ratio, *LAVI* left atrial volume index, *LV* left ventricular, *MI* myocardial infarction, *MR* mitral regurgitation, *MV* mitral valve, *PLAS* peak reservoir left atrial strain, *SI* sphericity index, *SVI* stroke volume index, *TAPSE* tricuspid annular plane systolic excursion

### Transthoracic echocardiography

Preoperative transthoracic echocardiography was conducted using Vivid-7 and Vivid-E9 ultrasound machines (GE Vingmed Ultrasound, Horton, Norway) and stored in the institutional data repository. Retrospective re-evaluation of echocardiography, including STE analysis, was performed for the purpose of this study. Cardiac chamber quantification was performed in accordance with recent guidelines [18]. The grade of LV diastolic dysfunction was estimated according to current guidelines based on transmitral flow by pulse-wave Doppler and, where available, additional parameters. According to these parameters, LV diastolic dysfunction was graded as first, second and third grade [4]. Only a few cases with assessment of early diastolic mitral annular velocity (e′) derived from tissue Doppler imaging were available. Cases where conventional evaluation of diastolic function using an integrative approach [4] was not possible due to the lack of parameters were excluded from the analysis.

### Speckle-tracking echocardiography

Speckle-tracking analysis was performed using EchoPac Software version 201 (GE Vingmed Ultrasound A/S, Norway). Longitudinal parameters of LV function were obtained from apical long axis, 4-chamber, and 2-chamber views by manual tracking of the endocardial border [19]. Only segments with satisfactory tracking through the entire cardiac cycle were included in the analysis. The median frame rate of images used for STE was 61 fps (IQR 55–70 fps). For all STE measurements, the R-peak at ECG was used as a reference point for the beginning of the cardiac cycle. Aortic valve closure as a reference for end-systole was manually adjusted in apical long axis view. The longitudinal parameters measured were end-systolic strain, peak systolic strain rate, and early and late diastolic strain rate (GLSRe and GLSRa). All parameters were averaged from the 18-segment LV model obtained from apical long axis, 4-, and 2-chamber views. In 99 patients (72.3%), all 18 segments could be evaluated by STE, in other 38 patients (27.7%) 12–17 segments were included in STE analysis. The median number of segments evaluated for STE per patient was 18 (IQR 17–18 segments) Additionally, STE parameters obtained at the basal LV level (averaged from 6 basal LV segments) were quantified and reported. For evaluation of left atrial (LA) longitudinal function, we measured the peak reservoir left atrial strain (PLAS) during the reservoir phase in the apical 4-chamber view, with R-peak as a reference point for the start of the cardiac cycle [20]. Additionally, the ratios of early diastolic filling velocity (E) to each diastolic STE parameter were calculated and reported.

### Hemodynamic measurements

Preoperative heart catheterization was performed for 27 patients within 6 days of echocardiography (median 2 days, IQR: 1–4 days). The parameters assessed were mean pulmonary artery pressure (PAP) in 20 patients, pulmonary capillary wedge pressure (PCWP) in 15 patients and LV end-diastolic pressure (LVEDP) in 24 patients.

### Statistical analysis

All variables are presented as the mean (± SD), median (with interquartile intervals), or percentage where applicable. Categorical variables are presented as numbers with percentages. Differences in baseline patient characteristics between patients undergoing cardiac catheterization compared and the total population were assessed using the unpaired* t*-test or the Mann–Whitney *U* test for continuous variables as appropriate, and by the chi-squared test for qualitative variables. Pearson correlation and regression analyses were performed to assess the association between STE parameters and hemodynamic parameters with the further application of receiver operating characteristic (ROC) analysis evaluating the accuracy of STE parameters to detect elevated values for hemodynamic parameters and to predict combined outcome. Optimal cut-offs were estimated based on the Youden equation [21]. Univariate and multivariate Cox proportional hazard regression analyses were applied to assess the association of preoperative clinical and echocardiographic parameters with the combined outcome. Individual echocardiographic parameters were evaluated separately adjusted for important clinical variables. Variables included in the model were selected on the basis of their clinical importance and their potential to confound the parameters of interest. To this end, a clinical model was initially run to evaluate the most important parameters associated with combined outcome. Stepwise addition of conventional echocardiographic parameters on the top of clinical model was performed and further addition of various STE parameters. Kaplan–Meier analysis was conducted to estimate the differences in event-free survival between the groups by cut-off for STE parameters.

Interobserver variability of STE parameters was assessed for 20 randomly selected studies by two independent echo investigators (ON and YH). The variability is expressed as the interclass correlation coefficient (ICC). Statistical significance was defined by *p* < 0.05. The data were analyzed using SPSS, version 25 (SPSS, Chicago, IL, USA).

## Results

### Patient characteristics

Baseline patient characteristics with univariate analysis for prediction of outcome are listed in Table 1. There was no significant difference in baseline clinical and echocardiographic parameters between patients who underwent heart catheterization and the total population (Suppl. Table 1).

### Association of diastolic STE parameters with hemodynamic measurements

**Fig. 2:**
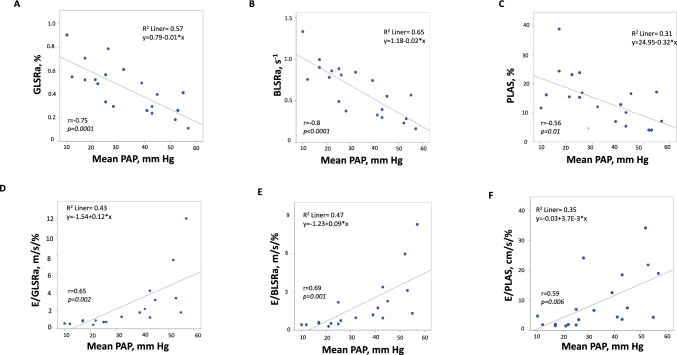
Association between STE parameters and mean PAP

**Fig. 3:**
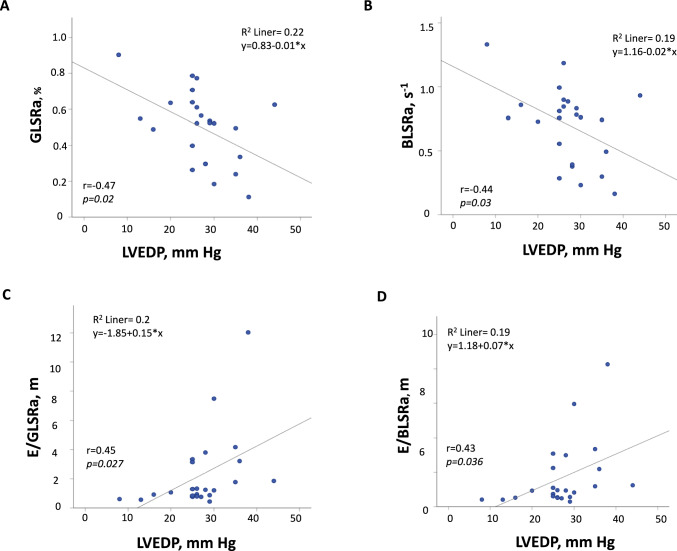
Association between STE parameters and LVEDP

The average mean PAP was 33.2 ± 15 mmHg, the average mean PCWP was 26.5 ± 11 mmHg and the average LVEDP was 27 ± 8 mmHg at heart catheterization. The correlation of echocardiographic parameters with hemodynamic measures is presented in Table 2. The correlation of “conventional” parameters—especially E/A—and the systolic strain rate, both global and basal, were significant. There was a significant correlation of PLAS, GLSRa and E/GLSRa with mean PAP and mean PCWP. Parameters obtained at the basal level of the left ventricle, such as BLSRa and E/BLSRa, demonstrated somewhat stronger associations with hemodynamic measures (Table 2, Fig. 2). A significant correlation of LVEDP with GLSRa, BLSRa, E/GLSRa and E/BLSRa was also observed (Table 2, Fig. 3).

**Table 2:** Correlation of echocardiographic parameters with hemodynamic measures

	Mean PAP *N* = 20		Mean PCWP *N* = 15		LV EDP *N* = 24	
	*r*	*p*-value	*r*	*p*-value	*r*	*p*-value
E, m/s	0.62	0.003	0.68	0.005	0.54	0.006
A, m/s	− 0.66	0.002	− 0.55	0.035	− 0.31	0.15
E/A	0.65	0.002	0.6	0.018	0.46	0.023
PLAS, %	− 0.56	0.01	− 0.61	0.015	− 0.21	0.33
E/PLAS, cm/s/%	0.59	0.006	0.75	0.026	0.33	0.12
GLS*, %	− 0.42	0.06	− 0.42	0.12	− 0.18	0.41
GLSRs*, s^−1^	− 0.52	0.02	− 0.6	0.02	− 0.32	0.13
GLSRe, s^−1^	− 0.17	0.48	− 0.47	0.08	− 0.1	0.64
GLSRa, s^−1^	− 0.75	0.0001	− 0.58	0.025	− 0.47	0.02
E/GLSRe, m	0.36	0.12	0.58	0.023	0.14	0.52
E/GLSRa, m	0.65	0.002	0.62	0.014	0.45	0.027
BLS*, %	− 0.52	0.018	− 0.57	0.028	− 0.31	0.15
BLSRs*, s^−1^	0.44	0.05	− 0.51	0.052	− 0.1	0.63
BLSRe, s^−1^	− 0.16	0.49	− 0.43	0.11	− 0.16	0.46
BLSRa, s^−1^	− 0.8	0.00002	− 0.65	0.009	− 0.44	0.03
E/BLSRe, m	0.55	0.013	0.71	0.003	0.35	0.1
E/BLSRa, m	0.69	0.001	0.64	0.01	0.43	0.036

*A* late diastolic filling velocity, *BLS* basal longitudinal strain, *BLSRa* basal late diastolic longitudinal strain rate, *BLSRe* basal early diastolic longitudinal strain rate, *BLSRs* basal systolic longitudinal strain rate, *GLS* global longitudinal strain, *GLSRa* global late diastolic longitudinal strain rate, *GLSRe* global early diastolic longitudinal strain rate, *GLSRs* global systolic longitudinal strain rate, *E* late diastolic filling velocity, *LVEDP* left ventricular end-diastolic pressure, *PAP* pulmonary artery pressure, *PCWP* pulmonary capillary wedge pressure, *PLAS* peak reservoir left atrial strain

*r* Pearson correlation coefficient

*Correlation performed for absolute values of STE parameters

**Fig. 4:**
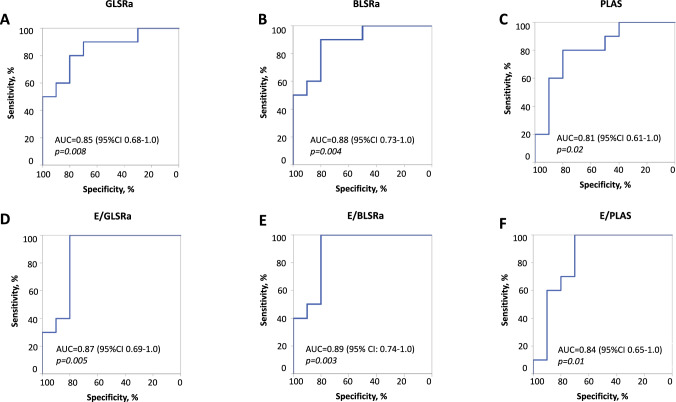
ROC curves for STE parameters to detect mean PAP ≥ 30 mmHg

We performed ROC analysis to assess the ability of the STE parameters to detect elevated mean PAP and LVEDP values. Higher threshold values for elevated mean PAP and LVEDP (≥ 30 mm Hg for mean PAP and ≥ 20 mm Hg for LVEDP) than recommended were used, as most patients presented with elevated intracardiac hemodynamic parameters. Left atrial strain, global and basal late diastolic strain rates, and the ratio of early diastolic filling velocity to these parameters demonstrated comparable specificity and sensitivity compared to Doppler parameters (Table 3, Fig. 4). Although there was a correlation between late diastolic strain rate parameters and LVEDP, only E/BLSRa demonstrated a predictive value to detect severely elevated LVEDP in ROC analysis (Suppl. Table 2).

**Table 3:** Sensitivity and specificity of echocardiographic parameters to detect mean PAP ≥ 30 mm Hg

	Optimal cut-off value*	Sensitivity, %	Specificity, %	AUC	95% CI	*p*-value
E, m/s	0.74	80	80	0.78	0.57–0.99	0.03
A, m/s	0.69	100	70	0.86	0.68–1.0	0.007
E/A	1.1	90	80	0.86	0.69–1.0	0.007
PLAS, %	12.8	80	80	0.81	0.61–1.0	0.02
E/PLAS, cm/s/%	3.7	100	70	0.84	0.65–1.0	0.01
GLSRa, s^−1^	0.41	80	80	0.85	0.68–1.0	0.008
E/GLSRa, m	1.24	100	80	0.87	0.7–1.0	0.005
BLSRa, s^−1^	0.74	90	80	0.88	0.73–1.0	0.004
E/BLSRa, m	0.94	100	80	0.89	0.74–1.0	0.003

*A* late diastolic filling velocity,* BLSRa* basal late diastolic longitudinal strain rate,* GLSRa* global late diastolic longitudinal strain rate,* E* late diastolic filling velocity,* PLAS* peak reservoir left atrial strain

*Optimal cut-off based on Youden equation

### Outcome data

Five patients died in the hospital within 30 days after surgery. During a median follow-up period of 4.7 years (IQR: 1.4–8.1 years), total combined events occurred in 59 patients (43.1%): 58 patients died (42.4%, including those patients died within 30 days after the surgery), 8 patients (5.8%) received an LVAD, and 2 patients (1.5%) underwent heart transplantation. Overall, the event-free survival rate at 1 year was 84.6% (95% CI 77.4–89.7%), that at 5 years was 69.4% (95% CI 60.3–76.9%), and that at 10 years was 47.2% (95% CI 36–57.5%). The median event-free survival time was 9.5 years (95% CI 7.5–11.5 years). Overall, the 1-year survival rate was 87.6% (95% CI 80.8–92.1%), the 5-year survival rate was 72.5% (95% CI 63.5–79.6%), and the 10-year survival rate was 48.3% (95% CI 37.1–58.7%). No difference in event-free survival was found between men and women in the Kaplan–Meier analysis (*log rank p* = *0.31*) or in the Cox regression analysis (age-adjusted HR for female sex 0.86; 95% CI 0.5–1.5; *p* = *0.6*).

**Fig. 5:**
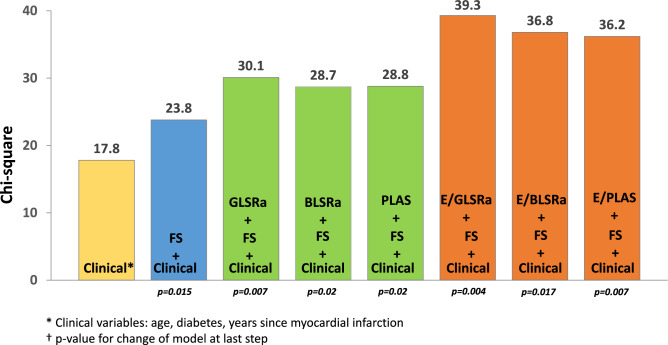
Incremental prognostic value of STE parameters. Adding STE parameters to the model consisting of important clinical variables (age, diabetes mellitus and time since MI) and echocardiographic measures of LV systolic function (fractional shortening) resulted in a substantial increase in the significance of the model

According to univariate Cox regression analysis, several conventional echocardiographic and STE parameters demonstrated an association with the combined outcome (Table 1). Clinical and echocardiographic multivariate models were run to identify variables to be included in the final model (Suppl. Table 3). The multivariate model, consisting of clinical parameters (age, diabetes mellitus, time since myocardial infarction) with stepwise addition of one echocardiographic parameter, fractional shortening (FS), and STE parameters of diastolic function on the top of the model, demonstrated incremental prognostic value of STE parameters for the association with an outcome (Fig. 5). E/GLSRa demonstrated the most significant improvement of the baseline model (χ^2^ increased from 23.8 for the model consisting of clinical factors and FS to 39.3 after adding E/GLSRa on the top of the model, *p* = 0.004). Although age did not demonstrate significance in the multivariate model, it was included in the final model, as age may confound other echocardiographic parameters.

**Fig. 6:**
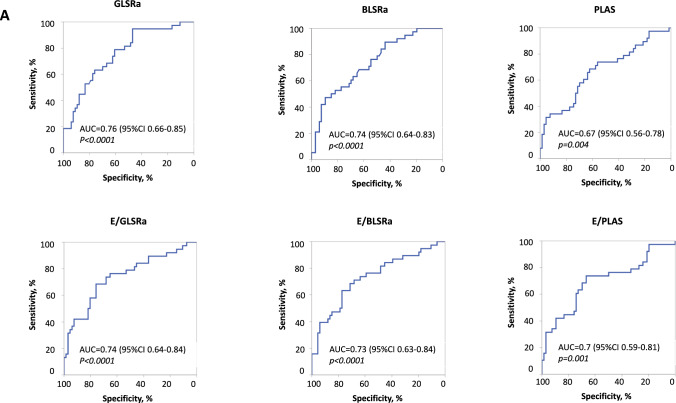
ROC curves for STE parameters to predict combined outcome

In the ROC analysis, left atrial strain, global and basal late diastolic strain rate, and the ratios of early diastolic filling velocity to these parameters were significantly associated with the combined 5-year outcome (Fig. 6). The cut-off values identified in ROC analysis differ from those cut-offs identified for the detection of mean PAP ≥ 30 mmHg (Tables 3, 4).

**Table 4:** Sensitivity and specificity of echocardiographic parameters to predict combined outcome at 5 year

	Optimal cut-off value*	Sensitivity, %	Specificity, %	AUC	95% CI	*p*-value
E, m/s	0.75	57.9	72.7	0.66	0.54–0.77	0.007
A, m/s	0.58	65.8	68.2	0.64	0.52–0.75	0.21
E/A	1.56	57.9	77.3	0.67	0.56–0.78	0.004
PLAS, %	16.9	68.4	62.1	0.67	0.56–0.78	0.004
E/PLAS, cm/s/%	4.1	73.7	66.7	0.7	0.59–0.81	0.001
GLSRa, s^−1^	0.59	94.7	46.9	0.76	0.66–0.85	< 0.0001
E/GLSRa, m	1.68	68.4	75.8	0.74	0.64–0.84	< 0.0001
BLSRa, s^−1^	0.41	47.4	89.4	0.74	0.64–0.83	< 0.0001
E/BLSRa, m	1.41	63.2	77.3	0.73	0.63–0.84	< 0.0001

*A* late diastolic filling velocity,* BLSRa* basal late diastolic longitudinal strain rate,* GLSRa* global late diastolic longitudinal strain rate,* E* late diastolic filling velocity,* PLAS* peak reservoir left atrial strain

*Optimal cut-off based on Youden equation

**Fig. 7:**
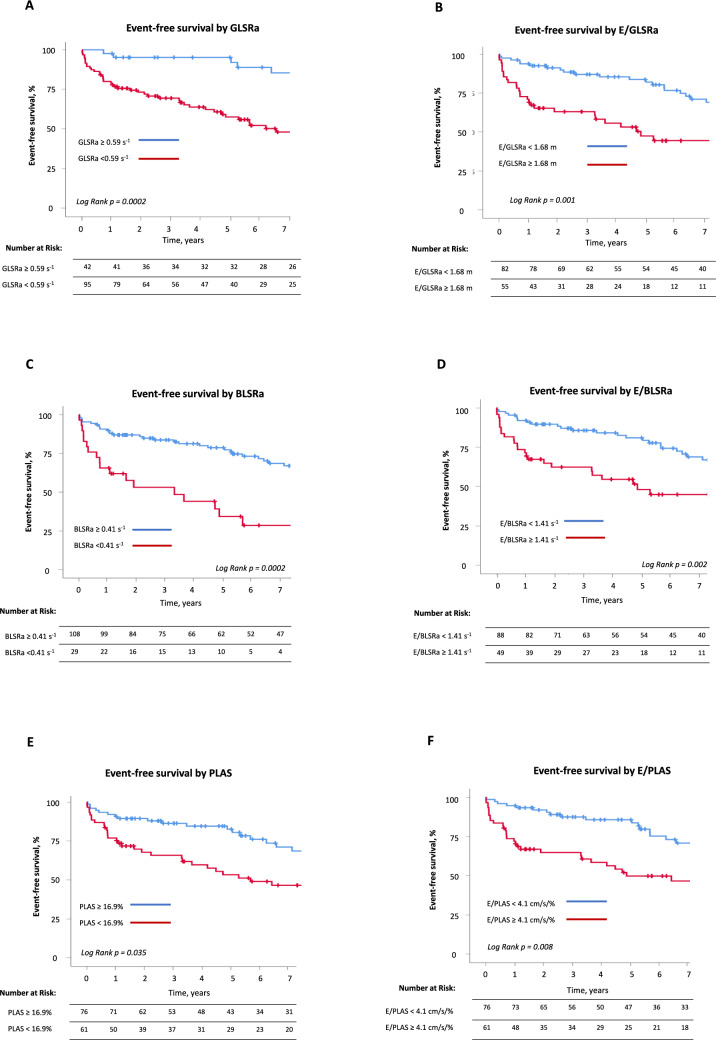
Event-free survival stratified by STE parameters

Patient stratification based on cut-off values for STE parameters to predict combined outcome revealed significant differences in event-free survival between groups. The clearest difference in survival was demonstrated for GLSRa. Kaplan–Meier analysis demonstrated that patients with GLSRa < 0.59 s^−1^ (*n* = 95) had significantly shorter event-free survival, with a mean survival time of 6.7 years (95% CI 5.6–7.8 years) compared to 10.9 years (95% CI 9.6–12.1 years) in those with GLSRa ≥ 0.59 s^−1^ (*n* = 42) (*p* = 0.0002) (Fig. 7, Panel A). Similarly, patients with BLSRa < 0.41 s^−1^ (*n* = 29) demonstrated shorter event-free survival than those with BLSRa ≥ 0.41 s^−1^ (*n* = 108), with a mean survival time of 5 years (95% CI: 3–7 years) vs. 9 years (95% CI 8–10 years), respectively (*p* = 0.0002) (Fig. 7, Panel C). PLAS ≥ 16.9% was associated with a longer mean event-free survival of 9.1 years (95% CI 7.9–10.2 years) compared to 7 years (95% CI: 5.5 to 8.5 years) for patients with PLAS < 16.9% (p = 0.035) (Fig. 7, Panel C). In contrast, higher values of E/GLSRa, E/BLSRa and E/PLAS were associated with a worse outcome (Fig. 7, Panels B, D and F).

### Reproducibility of LV speckle-tracking analysis

Good interobserver agreement was demonstrated for global longitudinal strain (ICC 0.96; 95% CI 0.89–0.98, *p* < 0.0001), GLSRe (ICC 0.94, 95% CI: 0.84–0.98, *p* < 0.0001), GLSRa (ICC 0.97, 95% CI 0.93–0.99, *p* < 0.0001), and PLAS (ICC 0.82, 95% CI 0.55–0.93, *p* = 0.0002).

## Discussion

The present study aimed to evaluate LV diastolic function in patients with LV aneurysm using conventional and speckle-tracking echocardiographic parameters. As a first step, we investigated the association of echocardiographic parameters with invasive cardiac hemodynamic measures. We found that the longitudinal late diastolic strain rate at global and basal levels of the left ventricle correlated more strongly with mean PAP than conventional transmitral flow parameters and was able to detect a significantly increased mean PAP in our group of patients. It also has a significant correlation with mean PCWP and LVEDP. Lower GLSRa and BLSRa were associated with higher values for mean PAP, mean PCWP and LVEDP. Left atrial strain also demonstrated a correlation with mean PAP and PCWP.

Furthermore, we evaluated STE parameters in the outcome analysis and found that late diastolic strain rate at the global and basal level of the left ventricle, left atrial strain and ratio of early diastolic filling velocity to these parameters showed an association with event-free survival after adjustment for age and sex, as well as in a multivariate model including clinical and echocardiographic parameters. The best predictive value was demonstrated for the ratio of early diastolic filling velocity to GLSRa.

### LV diastolic function by STE

Impaired LV diastolic function in patients with ischemic cardiomyopathy may be assessed using a similar integrative approach with the assessment of various echocardiographic indices, including STE parameters, such as strain rate during isovolumic relaxation and early diastolic strain rate alone or in conjunction with transmitral early diastolic filling velocity velocity [1]. Experimental studies have shown a strong association of these parameters with invasively measured LV filling [5, 22]. However, data on the assessment of diastolic function by LV diastolic strain rate in patients with ischemic cardiomyopathy are limited. In a study by Meluzin et al. [23], the late diastolic strain rate was significantly lower in patients with dilated cardiomyopathy than in controls, showed a significant correlation with PCWP and was able to detect elevated PCWP. In the same study, the early diastolic strain rate revealed no association with PCWP. Recently, PLAS has been proposed as a valuable parameter to assess LV diastolic dysfunction [1] and has been found to have predictive value in patients with heart failure with reduced ejection fraction [6].

We demonstrated that STE parameters such as longitudinal late diastolic strain rate at global and basal levels of the left ventricle and left atrial strain were significantly correlated with mean PAP and mean PCWP. Furthermore, GLSRa and BLSRa showed a stronger correlation with the mean PAP than with the transmitral flow parameters. Among other STE parameters, only GLSRa and BLSRa correlated with LVEDP. GLSRa, BLSRa, PLAS and the ratio of early diastolic filling velocity to these parameters were able to detect severely elevated mean PAP (≥ 30 mmHg), with the predictive values expressed as the area under the curve (AUC) ranging from 0.81 to 0.89 and comparable to the AUC for transmitral flow velocities.

SR_IVRT_ was not used in our study despite its proven role in the assessment of LV diastolic function [1, 5]. In our particular group of patients with severe LV remodeling, quantification of SR_IVRT_ appeared to be poorly reproducible; therefore, we decided to focus on other STE diastolic parameters.

### Prognostic role of LV diastolic function by echocardiography in patients with LV aneurysm

Previous studies have demonstrated that advanced diastolic dysfunction is associated with impaired survival after SVR [12, 22, 23]. A recent analysis of the STICH trial also confirmed that LV diastolic dysfunction assessed by the *E/A* ratio was a stronger predictor of survival than LV geometry assessed by the sphericity index [24]. In previous reports, diastolic dysfunction was evaluated using transmitral Doppler parameters [12, 23] or the more sensitive parameter *E/e*′ [22]. In one study, postoperative LV remodeling after SVR was evaluated using SR_IVRT_ and demonstrated a good association with LV filling pressure [25]. Neither PLAS nor diastolic LV strain rate were previously evaluated for preoperative assessment of patients with LV aneurysm planned for SVR. In our previous study, we found that basal LV strain was associated with outcome and with improvement of regional LV function after SVR [26]. In the present study, STE parameters of diastolic dysfunction, such as PLAS and longitudinal late diastolic strain rate, were predictors of event-free survival and demonstrated incremental predictive value after adjustment for clinical and echocardiographic parameters. The highest predictive value was demonstrated for echocardiographic indices: ratio of early diastolic filling velocity to GLSRa and BLSRa. In other studies, it was also shown that the combination of early diastolic filling velocity with diastolic parameters of LV mechanics is useful for the assessment of LV diastolic pressure and plays a predictive role in various groups of patients [5].

In this study, the early diastolic strain rate was not associated with hemodynamic measures or with outcome. These data are consistent with the study of Meluzin et al. [23], where the early diastolic strain rate was also not associated with PCWP in patients with dilated cardiomyopathy. While early diastolic parameters mainly reflect active relaxation of the LV, parameters of late diastole are related to LV compliance [1]. This might be explained by significantly increased stiffness and decreased compliance of the left ventricle at this stage of heart failure.

### Future perspectives

Recent advances in echocardiography allow automated quantification of LV myocardial mechanics using the speckle-tracking technique. This opens future opportunities for complex automated analysis of LV systolic and diastolic function, which could also be implemented in point-of-care ultrasound [27]. Our results demonstrate that the use of STE to assess LV diastolic function can play a prognostic role and might be useful in the preoperative evaluation of patients planned for SVR. STE quantification does not require a high level of expertise or additional image acquisition, and the analysis can be performed offline and has a low inter-observer variability, as demonstrated by our data and previous studies [28].

Assessment of additional diastolic parameters with STE may play important role in the integrative evaluation of LV diastolic function and become a part of a complex integrative approach, particularly in patients with ischemic cardiomyopathy. Its diagnostic and prognostic role should be further evaluated in different populations.

STE can be used for postsurgical assessment of LV diastolic function in patients with various pathologies. For this purpose, prospective studies are needed.

### Limitations

It should be acknowledged that the current study has certain limitations. First, by its nature, it is subject to the restrictions of a nonrandomized single-center observational retrospective study. Only patients with feasible data were included; therefore, selection bias cannot be overcome. Women were underrepresented. Furthermore, there was a limited number of patients with tissue Doppler for quantification of early mitral annulus velocity. Thus, these data are not available for comparison. We included in the analysis those patients for whom the evaluation of diastolic function without e′ was possible based on other echocardiographic parameters.

Only a small number of patients underwent cardiac catheterization. Although there was no statistically significant difference in clinical and echocardiographic parameters between this group of patients and total cohort, there was a tendency for more deterioration in systolic and diastolic LV function and greater cardiac remodeling in patients who underwent catheterization. We believe that this group can be representative to demonstrate the association of STE parameters of diastolic function and heart hemodynamics and that the data can be generalized to a larger population of patients with post-ischemic LV aneurysm.

At the time of data collection, STE was already a standard technique in our institution, and 2-dimensional echocardiography was performed according to the highest level of expertise. All echocardiographic data were acquired using a single vendor ultrasound machine; thus, the cutoff values for STE parameters may be different for other vendors.

## Conclusion

In patients with LV aneurysms, late diastolic strain rate and left atrial strain can be used to assess LV diastolic function. Evaluation of LV diastolic function with STE-derived parameters plays an important role in predicting outcome in patients with LV aneurysms and should be considered in the preoperative evaluation. The incorporation of these parameters into the preoperative evaluation of patients planned for SVR may guide the decision-making process. Furthermore, the results of this study might be used for the further development of automated and semiautomated analyses of LV function. Future studies should prospectively investigate the impact of STE.

## Supplementary Information

Below is the link to the electronic supplementary material.

Supplementary file1 (DOCX 19 KB)

Supplementary file2 (DOCX 17 KB)

Supplementary file3 (DOCX 16 KB)

## Abbreviations

AUC: Area under the curve
BLSRa: Basal late longitudinal diastolic strain rate
E: Early diastolic filling velocity
EF: Ejection fraction
FS: Fractional shortening
GLSRa: Global late longitudinal diastolic strain rate
GLSRe: Global early longitudinal diastolic strain rate
ICC: Interclass correlation coefficient
LV: Left ventricular
LVAD: Left ventricular assist device
LVEDP: Left ventricular end-diastolic pressure
PAP: Pulmonary artery pressure
PCWP: Pulmonary capillary wedge pressure
PLAS: Peak reservoir left atrial strain
ROC: Receiver operating characteristic
STE: Speckle-tracking echocardiography
SVR: Surgical ventricular restoration
SR_IVRT_: Longitudinal diastolic strain rate measured at isovolumic relaxation time
