# Supplementary material for: Left ventricular diastolic function assessed by speckle tracking echocardiography in patients with left ventricular aneurysm
Source: Int J Cardiovasc Imaging. 2024 Jul 25;40(10):2087–101. doi: 10.1007/s10554-024-03201-z (PMC11499540; doi:10.1007/s10554-024-03201-z)
Supplement: Supplementary file 1 — Supplementary file1 (DOCX 19 KB) [file 10554_2024_3201_MOESM1_ESM.docx]

**Supplemental Table 1. Clinical characteristics of patients for whom heart catheterization was performed.**

| **Parameter** | **Patients with catheter**  **N=27** | **All patients**  **N=137** | ***p*-value** |
| --- | --- | --- | --- |
| Age, years | 59.9±11 | 60.8±11 | 0.73 |
| Women/Men | 6/21 (22.2/77.8) | 34/103 (24.8/75.2) | 0.15 |
| BMI, kg/m^2^ | 26 [24; 30] | 27 [24; 31] | 0.29 |
| Diabetes mellitus | 11 (40.7) | 36 (26.3) | 0.2 |
| Arterial hypertension | 17 (63) | 98 (71.5) | 0.51 |
| Paroxysmal atrial fibrillation | 5 (18.5) | 13 (9.5) | 0.3 |
| Chronic kidney disease, stage ≥3 | 3 (11.1) | 25 (18.2) | 0.58 |
| Plasma creatinine, mg/dL | 1.17 [0.91; 1.36] | 1.02 [0.87; 1.27] | 0.14 |
| NYHA functional class III-IV | 26 (96.3) | 122 (89.1) | 0.48 |
| Time since MI, years | 6 [0.4; 11.7] | 2.8 [0.3; 12.6] | 0.75 |
| Previous heart surgery | 3 (11.1) | 8 (5.8) | 0.39 |
| **Echocardiography** |  |  |  |
| LV EDDI, cm/m^2^ | 3.1 [2.7; 3.5] | 3.0 [2.7; 3.4] | 0.59 |
| LV ESDI, cm/m^2^ | 2.4 [1.9; 2.7] | 2.4 [2.0; 2.7] | 0.51 |
| LV FS, % | 22 [14; 29] | 23 [15; 29] | 0.63 |
| LV EDVI, mL/m^2^ | 121 [96; 155] | 104 [84; 134] | 0.11 |
| LV ESVI, mL/m^2^ | 89 [64; 112] | 69 [53; 96] | 0.1 |
| LV EF, % | 31±9 | 34±9 | 0.11 |
| LAVI, mL/m^2^ | 43 [33; 55] | 41 [33; 53] | 0.7 |
| Diastolic dysfunction, grade 3 | 10 (37) | 46 (33.6) | 0.9 |
| MR 2+ | 10 (37) | 35 (25.5) | 0.32 |
| GLS, % | -4.4±3 | -5.5±3 | 0.11 |
| PLAS, % | 14.8 [8.6; 19.7] | 17.9 [12; 24] | 0.11 |

Data are presented as the mean ± SD, median [1. and 3. quartiles], or n (%).

Abbreviations: GLS – global longitudinal strain, EDDI – end-diastolic diameter index, EDVI – end-diastolic volume index, EF – ejection fraction, ESDI – end-systolic diameter index, ESVI – end-systolic volume index, FS – fractional shortening, LV – left ventricular, LAVI – left atrial volume index, MI – myocardial infarction, MR – mitral regurgitation, PLAS – peak reservoir left atrial strain,.
