# Supplementary material for: Left ventricular diastolic function assessed by speckle tracking echocardiography in patients with left ventricular aneurysm
Source: Int J Cardiovasc Imaging. 2024 Jul 25;40(10):2087–101. doi: 10.1007/s10554-024-03201-z (PMC11499540; doi:10.1007/s10554-024-03201-z)
Supplement: Supplementary file 2 — Supplementary file2 (DOCX 17 KB) [file 10554_2024_3201_MOESM2_ESM.docx]

**Supplemental Table 1. Data of ROC analysis of diastolic echocardiographic parameters to detect mean LVEDP ≥20 mm Hg.**

|  | Optimal cut-off value* | Sensitivity, % | Specificity, % | AUC | 95% CI | *p-value* |
| --- | --- | --- | --- | --- | --- | --- |
| E, m/s | - | - | - | 0.78 | 0.57 - 0.99 | 0.09 |
| A, m/s | - | - | - | 0.63 | 0.33 – 0.92 | 0.44 |
| E/A | - | - | - | 0.74 | 0.53 – 0.95 | 0.14 |
| PLAS, % | - | - | - | 0.59 | 0.34 – 0.83 | 0.59 |
| E/PLAS, cm/s/% | - | - | - | 0.69 | 0.46 – 0.92 | 0.12 |
| GLSRa, s^-1^ | - | - | - | 0.71 | 0.46 – 0.97 | 0.13 |
| E/GLSRa, m | - | - | - | 0.81 | 0.62 - 1.0 | 0.05 |
| BLSRa, s^-1^ | - | - | - | 0.66 | 0.38 – 0.93 | 0.14 |
| E/BLSRa, m | 0.55 | 85 | 75 | 0.84 | 0.65 - 1.0 | 0.036 |

Abbreviations: A - late diastolic filling velocity, AUC – area under the curve, BLSRa – basal late diastolic longitudinal strain rate, GLSRa – global late diastolic longitudinal strain rate, E – late diastolic filling velocity, LVEDP – left ventricular end-diastolic pressure, PLAS – peak reservoir left atrial strain.

*Optimal cut-off based on Youden equation
