# Supplementary material for: Left ventricular diastolic function assessed by speckle tracking echocardiography in patients with left ventricular aneurysm
Source: Int J Cardiovasc Imaging. 2024 Jul 25;40(10):2087–101. doi: 10.1007/s10554-024-03201-z (PMC11499540; doi:10.1007/s10554-024-03201-z)
Supplement: Supplementary file 3 — Supplementary file3 (DOCX 16 KB) [file 10554_2024_3201_MOESM3_ESM.docx]

**Supplemental Table 2. Clinical and echocardiographic multivariate models for the association with combined outcome.**

| **Parameter** | **HR** | **95% CI** | **p-value** |
| --- | --- | --- | --- |
| **Clinical model** |  |  |  |
| Age, 10 years | 1.1 | 0.8-1.5 | 0.51 |
| Diabetes | 2.2 | 1.1-4.3 | 0.02 |
| AF | 2.2 | 1.1-4.3 | 0.1 |
| Creatinine, 0.1 mg/dL | 1.05 | 1.0-1.1 | 0.055 |
| Time since MI, years | 1.05 | 1.01-1.1 | 0.01 |
| **Echocardiographic model** |  |  |  |
| LV ESVI, 10 mL/m^2^ | 0.9 | 0.8-1.05 | 0.23 |
| LV FS, 5 % | 0.8 | 0.7-0.96 | 0.014 |
| Shape, type 3 | 1.4 | 0.9-1.9 | 0.1 |
| Diastolic dysfunction grade | 1.1 | 0.8-1.5 | 0.74 |
| LAVI, 10 ml/m^2^ | 1.1 | 0.9-1.4 | 0.24 |
| LV mass index, 20 ml/m^2^ | 1.1 | 0.9-1.3 | 0.19 |

Abbreviations: AF – atrial fibrillation, MI – myocardial infarction, LAVI – left atrial volume index, LV – left ventricular, ESVI – end-systolic volume index, FS – fractional shortening,
